# Comparison of Jailed Wire and Jailed Balloon for Prevention of Side Branch Occlusion in Provisional Stenting: Evidence from a Systematic Review and Meta-Analysis

**DOI:** 10.31083/j.rcm2503107

**Published:** 2024-03-15

**Authors:** Dongdong Li, Hao Liu, Huimiao Dai, Chuncheng Gao, Pei Yang, Wangang Guo

**Affiliations:** ^1^Department of Cardiology, The Second Affiliated Hospital of Air Force Medical University, 710038 Xi’an, Shaanxi, China

**Keywords:** bifurcation, jailed balloon technique, jailed wire technique, meta-analysis, provisional stenting

## Abstract

**Background:**

Side branch (SB) occlusion after main vessel stenting is the main complication in treating coronary bifurcation lesions by provisional stenting. The Jailed Wire Technique (JWT), recommended by the European Bifurcation Club, is a standard technique to deal with this issue. The Jailed Balloon Technique (JBT) has been found to be more effective than the JWT in clinical practice by some interventionists, but it has not been widely accepted. In this meta-analysis, we compared the efficacy and safety of JBT and JWT.

**Methods:**

The literature comparing JBT and JWT was systematically reviewed. Stata/MP 17.0 was used to perform a meta-analysis. The primary endpoints were major adverse cardiac events (MACE), cardiac death, myocardial infarction (MI) and target lesion revascularization (TLR). The secondary endpoints were SB occlusion and SB dissection. Aggregated odds ratios and 95% confidence intervals were calculated. A sensitivity analysis was conducted if $I^{2}$ was >50% or *p* < 0.01.

**Results:**

Thirteen studies involving 1789 patients were enrolled. JBT was found to have a significantly lower incidence of MACE, SB occlusion and dissection. The incidence of cardiac death, MI and TLR were also lower in the JBT group, though the differences were not significant.

**Conclusions:**

JBT prevents SB occlusion more effectively and does not increase immediate or long-term complications. JBT, or its modified versions, can be used to treat SBs with a high risk of occlusion.

## 1. Introduction

Side branch (SB) occlusion after main vessel stenting is the main complication during treatment of coronary bifurcation lesions by provisional stenting. The Jailed Wire Technique (JWT) has been recommended by the European Bifurcation Club (EBC) as a standard technique to deal with this issue [1]. However, data from several studies revealed that the efficacy of JWT was limited. In most cases, the jailed wire only acted as a marker or path to rescue the compromised SB [2]. Burzotta* et al*. [3] first proposed the Jailed Balloon Technique (JBT) in 2010, in which a small balloon was jailed in place of a guidewire. With the JBT, the incidence of SB occlusion could be significantly reduced due to the larger spatial occupation. However, to the best of our knowledge, JBT increased the risk of vessel dissection especially in the SB ostium. There is currently no clear evidence as to whether JBT or JWT should be adopted when performing provisional stenting. This systematic review and meta-analysis sought to clarify this issue by comparing the immediate procedural outcomes and long-term follow-up of these two techniques. This meta-analysis was performed in accordance with the Preferred Reporting Items for Systematic Reviews and Meta-analysis and the Cochrane Collaboration guidelines, and has been registered on https://inplasy.com/ (*INPLASY202310082*).

## 2. Methods

### 2.1 Literature Searching Strategy

A systematic literature search was performed with PubMed, Ovid Medline, Web of Science, Embase, Cochrane, CNKI, Wanfang and Weipu from the databases inception to January 2023. The search items and strategy were “jailed balloon” or “jailed wire” and “bifurcation”. All relevant references were evaluated for addition once they met the inclusion criteria.

### 2.2 Literature Inclusion and Exclusion Criteria

Inclusion criteria included: (1) randomized controlled trials (RCTs) or observational studies comparing the JBT and JWT; (2) studies using drug-eluting stents.

Exclusion criteria included: (1) studies with unclear descriptions of the techniques or endpoints; (2) studies without the specified endpoints; (3) studies of low quality assessed by two independent reviewers; (4) repeated studies; (5) studies whose full text couldn’t be retrieved; (6) conference papers.

### 2.3 Endpoints

Primary endpoints: major adverse cardiac events (MACE) and its individual components including cardiac death, myocardial infarction (MI), target lesion revascularization (TLR).

Secondary endpoints: (1) SB occlusion defined as flow less than thrombolysis in MI (TIMI) 3; (2) SB vessel dissection, detected by angiography, optical coherence tomography or intravascular ultrasound.

### 2.4 Data Extraction and Quality Assessment

DL and HL extracted the data from the enrolled literature and independently assessed the qualities. Any conflict was resolved through discussions with HD. The extracted data included baseline characteristics of the enrolled studies and participants, and specified outcomes. The quality of RCTs was assessed with the Cochrane Collaboration tool [4]. The quality of observational studies was assessed with the Newcastle-Ottawa Quality Assessment Scale [5]. Any literature assessed as low quality by two reviewers was excluded.

### 2.5 Statistical Analysis

Aggregated odds ratios (OR) at 95% confidence intervals were calculated with Stata/MP 17.0 (Lakeway Drive, College Station, TX, USA). Heterogeneity between the studies was explored using the $I^{2}$ test. The random-effects model was used when *p* < 0.01 or $I^{2}$ > 50%, if not, the fixed-effects model was used. A heterogeneity test and sensitivity analysis were performed to select the origin of heterogeneity. Funnel plots and a regression-based Egger test were used to assess publication bias. The difference was considered statistically significant for a *p*-value < 0.05.

## 3. Results

### 3.1 Searching Results and Baseline Information

**Fig. 1.**
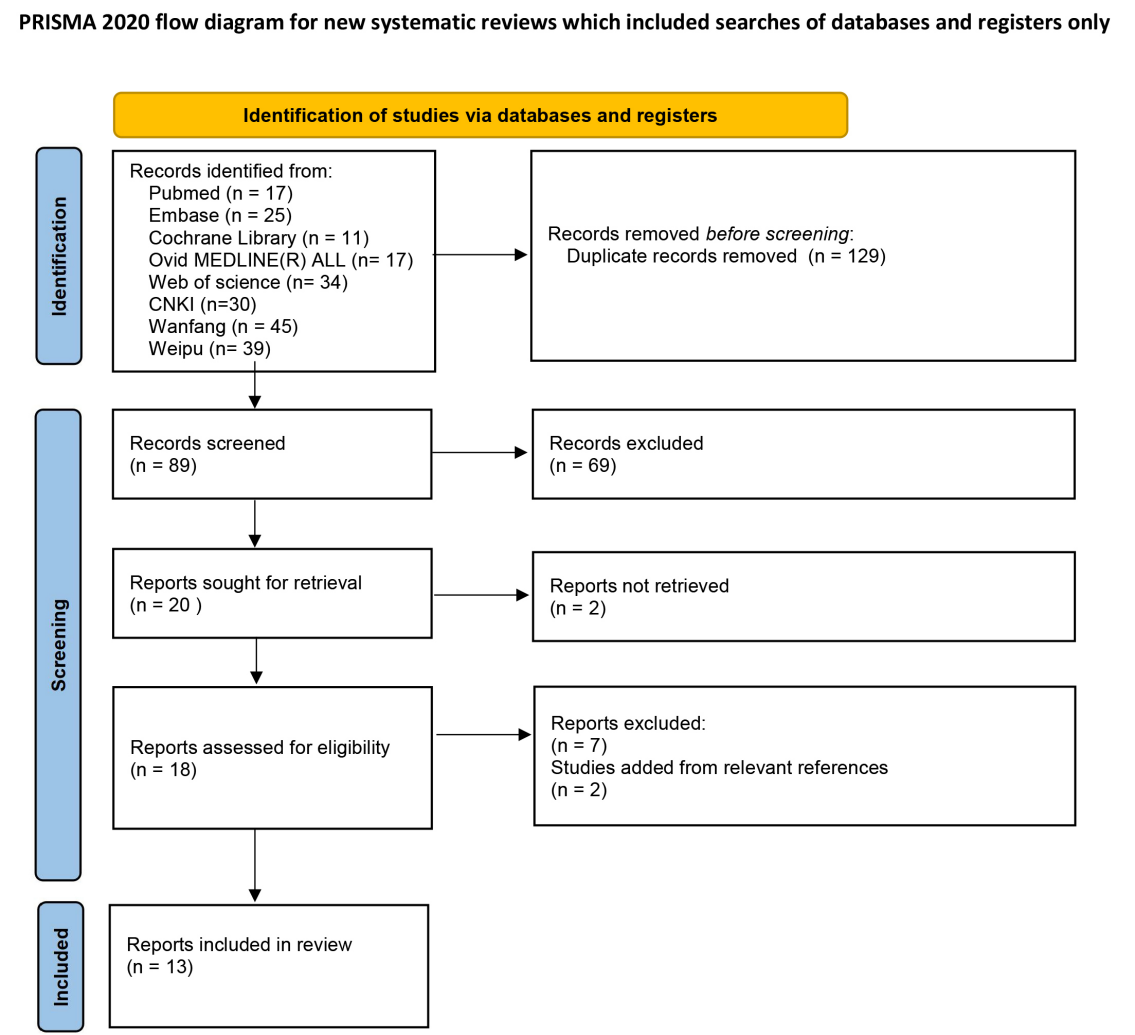
** Literature retrieval process**.

Eight databases were searched. From the 218 identified studies, 129 were excluded for being duplicates, 69 were excluded for not meeting the inclusion criteria, two were excluded for not retrieving the full text, and seven were excluded for meeting the exclusion criteria. Two were added by reviewing the relevant references. Finally, 13 studies involving 1789 patients were enrolled, including four published in English and nine published in Chinese [6, 7, 8, 9, 10, 11, 12, 13, 14, 15, 16, 17, 18]. The flowchart for identifying these studies is shown in Fig. 1.

The baseline information of the enrolled studies and participants is displayed in Tables 1A,1B,2 (Ref. [6, 7, 8, 9, 10, 11, 12, 13, 14, 15, 16, 17, 18]). There are nine RCTs and four observational studies. The publication year ranges from 2010 to 2020.

**Table 1A.** **Baseline characteristics of the enrolled studies and subjects**.

Study	Study type	Study period	Follow-up period	Number of patients (JWT/JBT)	Number of lesions (JWT/JBT)	Male, % (JWT/JBT)	Age, years (JWT/JBT)	History of PCI (JWT/JBT)
Jin, 2019 [6]	RCT	Jan.2013–May.2015	19.0 ± 6.1 months	45/44	45/45	66.7%/70.5%	65.4 ± 10.4/66.0 ± 8.8	6.7%/9.1%
Zhang, 2022 [7]	RCT	Dec.2016–Apr.2019	12.0 months	141/143	141/143	68.1%/74.8%	60.9 ± 10.0/61.1 ± 9.1	18.4%/14.0%
Qu, 2019 [17]	Non-RCT	Jan.2015–Jul.2017	12.0 months	80/40	80/40	77.5%/75%	59.5/62	17.5%/15%
Wang, 2021 [8]	RCT	May.2015–Dec.2019	-	216/216	216/216	75.46%/71.76%	57.28 ± 8.90/56.90 ± 8.90	-
Zeng, 2021 [10]	Non-RCT	Jan.2019–Mar.2020	-	30/30	30/30	70.0%/76.7%	55.7 ± 9.6/56.1 ± 9.4	-
Sun, 2018 [11]	RCT	Sep.2010–May.2015	12.0 months	40/41	40/41	62.5%/68.3%	51.9 ± 4.65/51.6 ± 4.15	-
Jian, 2020 [12]	Non-RCT	Jan.2016–Jun.2018	12.0 months	48/39	48/39	62.5%/59.0%	51.3 ± 16.5/59.3 ± 16.8	16.7%/15.4%
Guo, 2021 [14]	RCT	Jul.2019–Aug.2020	-	42/42	42/42	54.8%/59.5%	56.05 ± 9.87/55.18 ± 10.29	-
Han, 2018 [16]	RCT	Jun.2014–Jun.2017	12.0 months	128/128	128/128	51.6%/52.3%	65 ± 4.8/63 ± 5.2	-
Liang, 2021 [9]	RCT	Mar.2017–Oct.2018	12.0 months	48/48	48/48	54.2%/58.3%	57.37 ± 7.49/61.17 ± 8.15	-
Yang, 2019 [13]	RCT	Jan.2016–Mar.2018	6.0 months	30/30	30/30	60.0%/66.7%	64.5 ± 10.1/63.1 ± 11.2	-
Chen, 2021 [15]	RCT	Jan.2019–May.2020	12.0 months	30/30	30/30	40.0%/56.7%	66.23 ± 2.24/67.21 ± 3.37	-
Lai, 2018 [18]	Non-RCT	Jan.2014–Jan.2017	6.0 months	20/60	20/60	-	-	-

JWT, jailed wire technique; JBT, jailed balloon technique; RCT, randomized controlled trails; PCI, percutaneous coronary intervention.

**Table 1B.** **Continued**.

Hypertension (JWT/JBT)	Diabetes mellitus (JWT/JBT)	Hyperlipidemia (JWT/JBT)	Smoker (JWT/JBT)	Medina classification (JWT/JBT)	Lesion location (JWT/JBT)	Diagnosis (JWT/JBT)	Stent type (JWT/JBT)
73.3%/56.8%	35.6%/40.9%	28.9%/25.0%	37.8%/50.0%	1.1.1 64.4%/60.0%	LAD 75.6%/82.2%	SA or UA 51.1%/61.4%	Rapamycin-eluting stent 71.1%/80.0%
				1.0.1 13.3%/20.0%	LCX 8.9%/11.1%	NSTEMI 31.1%/15.9%	Paclitaxel-eluting stent 26.7%/20.0%
				0.1.1 22.2%/20.0%	RCA 15.6%/6.7%	STEMI 17.8%/22.7%	Adjuvant tamoxifen-eluting stent 2.2%/0%
						Chronic MI 13.3%/9.1%	
66.0%/56.6%	29.8%/28.0%	40.4%/39.2%	42.6%/46.2%	1.0.0 2.1%/2.1%	LAD 83.0%/85.3%	UA 53.9%/67.1%	-
				0.1.0 1.4%/1.4%	LCX 13.5%/12.6%		
				1.1.0 4.3%/7.7%	RCA 3.5%/2.1%		
				1.1.1 63.1%/62.2%			
				0.0.1 0%/0%			
				1.0.1 11.3%/10.5%			
				0.1.1 17.7%/16.1%			
52.5%/70%	23.8%/35%	31.3%/35%	48.8%/37.5%	1.1.1 78.8%/77.5%	LAD-D1 70.0%/67.2%	STEMI 13.8%/7.5%	-
				1.0.1 11.25%/12.5%	LAD-LCX 16.3%/17.5%	NSTEMI 27.5%/22.5%	
				0.1.1 10.0%/10.0%	LCX-OM 8.8%/5.0%	Angina 58.8%/70%	
					LAD-RI 1.3%/7.5%		
					RCA-PD 3.8%/2.5%		
61.11%/58.33%	33.80%/30.56%	95.83%/93.98%	-	1.0.0, 1.1.0, 0.1.0 22.69%/19.91%	LAD 72.22%/79.17%	-	-
				1.0.1, 1.1.1, 0.1.1 77.31%/80.09%	LCX 19.44%/12.04%		
					RCA 8.33%/8.80%		
66.7%/60.0%	43.3%/40.0%	50.0%/50.0%	43.3%/40.0%	-	-	-	-
90.0%/92.7%	45.0%/41.5%	72.5%/68.3%	52.5%/48.8%	-	-	-	Sirolimus-eluting stent 100.0%/100.0%
64.6%/61.5%	43.8%/35.9%	77.1%/69.2%	52.1%/48.7%	-	LAD 47.9%/56.4%	Prior MI 14.5%/12.8%	-
					LCX 18.8%/12.8%		
					RCA 33.3%/30.8%		
28.6%/35.7%	33.3%/21.4%	16.7%/23.8%	-	-	-	-	-
56.3%/58.6%	28.1%/31.0%	32.8%/35.05%	-	-	LAD/DB 25.8%/26.6%	SA 43.0%/45.3%	-
					LCX/OM 10.2%/10.9%	ACS 20.1%/22.2%	
-	-	-	-	-	-	-	-
-	-	-	-	-	-	-	-
66.7%/70.0%	33.3%/30.0%	-	-	-	-	-	-
-	-	-	-	-	-	-	-

LAD, left anterior descending coronary artery; LCX, left circumflex coronary artery; RCA, right ircumflex coronary artery; OM, obtuse marginal branch; SA, stable angina; UA, unstable angina; STEMI, ST segment elevation myocardial infarction; NSTEMI, non-ST segment elevation myocardial infarction; ACS, acute coronary syndromes; MI, myocardial infarction; RI, ramus medianus; PD, posterior descending branch; JWT, jailed wire technique; JBT, jailed balloon technique.

**Table 2.** **The specification of jailed balloon technique in each study**.

Study	JBT specification
Jin, 2019 [6]	Jailed balloon and stent balloon inflated simultaneously
Zhang, 2022 [7]	The jailed balloon was inflated only when necessary
Qu, 2019 [17]	Jailed balloon and stent balloon inflated simultaneously
Wang, 2021 [8]	The jailed balloon was inflated routinely after dilating the stent
Zeng, 2021 [10]	The jailed balloon was inflated only when necessary after dilating the stent
Sun, 2018 [11]	Jailed balloon and stent balloon inflated simultaneously
Jian, 2020 [12]	The jailed balloon was inflated routinely after dilating the stent
Guo, 2021 [14]	Jailed balloon and stent balloon inflated simultaneously
Han, 2018 [16]	Jailed balloon and stent balloon inflated simultaneously
Liang, 2021 [9]	The jailed balloon was inflated only when necessary after dilating the stent
Yang, 2019 [13]	-
Chen, 2021 [15]	Jailed balloon and stent balloon inflated simultaneously
Lai, 2018 [18]	The jailed balloon was inflated only when necessary after dilating the stent/Jailed balloon and stent balloon inflated simultaneously

JBT, jailed balloon technique.

### 3.2 Quality Assessment

**Fig. 2.**
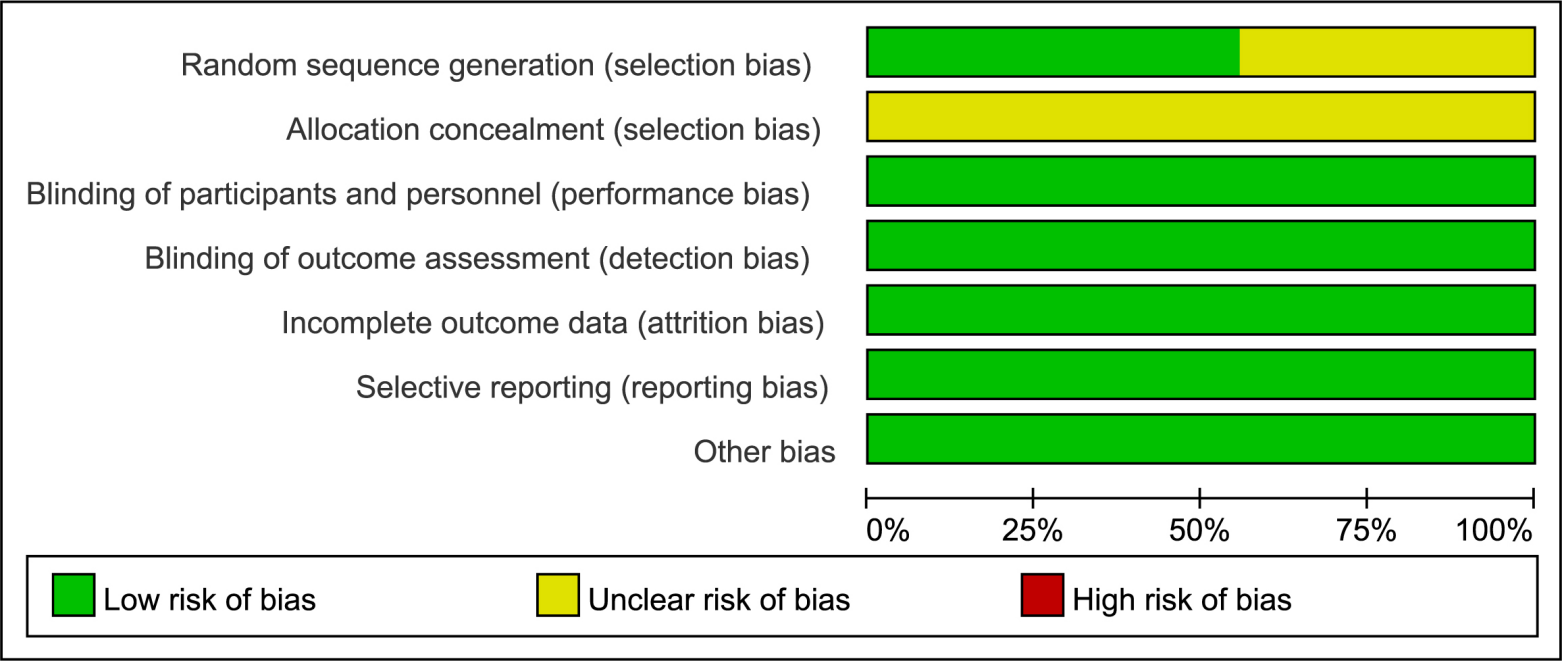
**Quality assessment of the RCTs with Cochrane Collaboration’s tool**. RCTs, randomized controlled trials.

The quality assessments of the RCTs are presented in Fig. 2. The quality assessments of the observational studies are shown in Table 3 (Ref. [10, 12, 17, 18]).

**Table 3.** **Quality assessment of the observational studies by NOS**.

Study	Selection	Comparability	Outcome	Total score
Qu, 2019 [17]	★★★	★★	★★★	8
Zeng, 2021 [10]	★★★	★★	★★★	8
Jian, 2020 [12]	★★★	★★	★★★	8
Lai, 2018 [18]	★★★	★★	★★★	8

NOS, Newcastle-Ottawa Quality Assessment Scale.

### 3.3 Primary Endpoints

#### 3.3.1 MACE 

**Fig. 3.**
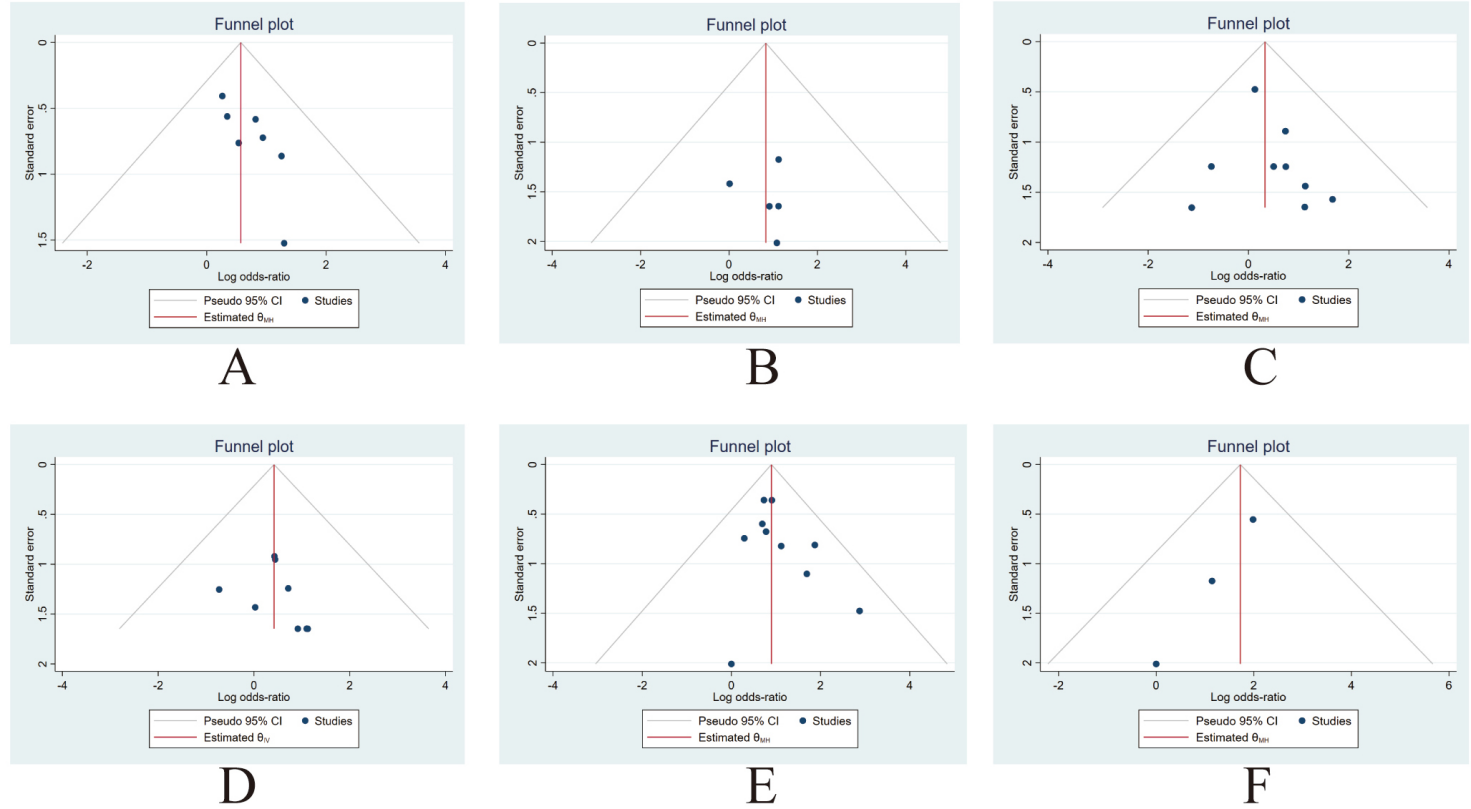
**Funnel plot for publication bias evaluation of MACE (A), cardiac death (B), MI (C), TLR (D), SB occlusion (E), and SB dissection (F)**. MACE, major adverse cardiac events; MI, myocardial infarction; TLR, target lesion revascularization; SB, side branch.

**Fig. 4.**
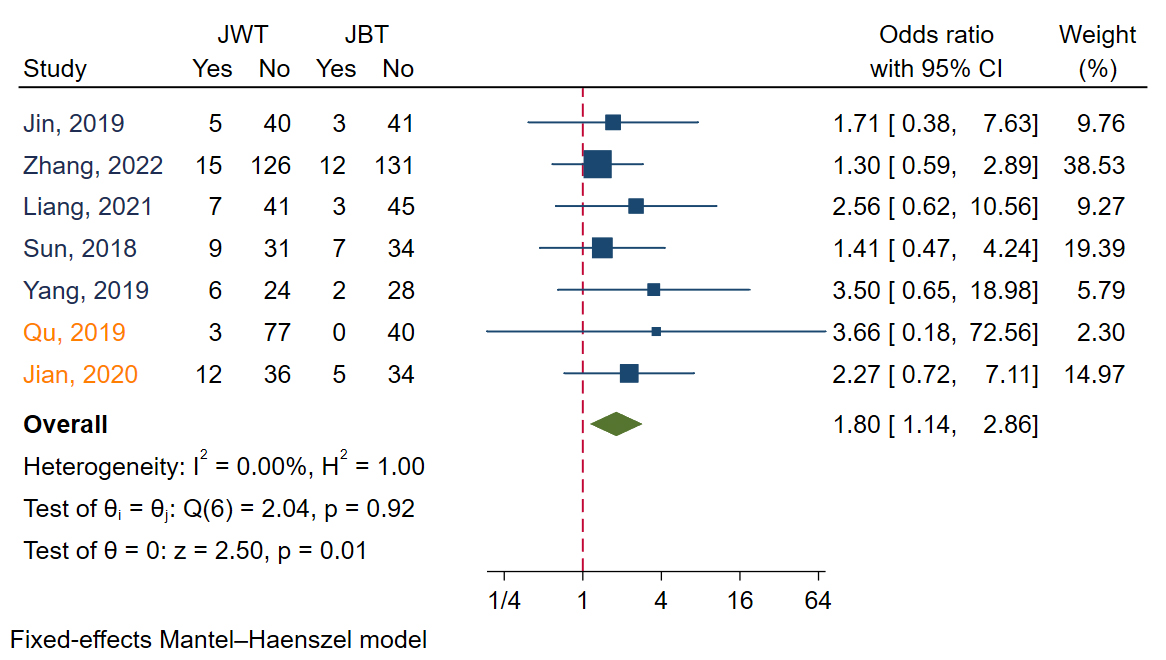
**Forest plot for comparisons of MACE between JBT and JWT**. MACE, major adverse cardiac event; JBT, jailed balloon technique; JWT, jailed wire technique. Studies in blue font are randomized controlled trails, while those in orange font are observational studies.

MACE was reported in seven studies [6, 7, 9, 11, 12, 13, 17]. $I^{2}$ value was 0.00, indicating that heterogeneity between these studies was small. Funnel plots and regression-based Egger test showed no evidence of publication bias (*p* = 0.26) (Fig. 3A). Meta analysis with a fixed model revealed that JBT had a significantly lower MACE than JWT (*p* = 0.01). The aggregated OR value was 1.80 (Fig. 4).

#### 3.3.2 Cardiac Death

**Fig. 5.**
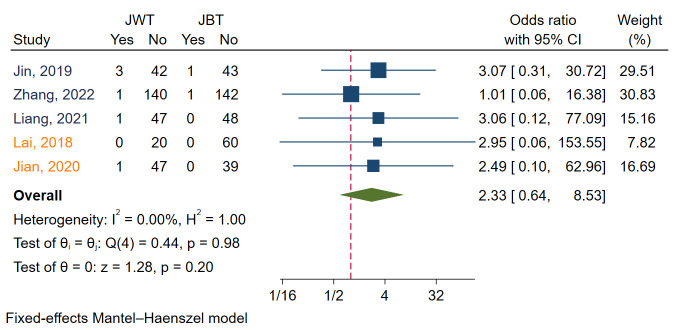
**Forest plot for comparisons of cardiac death between JBT and JWT**. JBT, jailed balloon technique; JWT, jailed wire technique. Studies in blue font are randomized controlled trails, while those in orange font are observational study.

Cardiac death was reported in five studies [6, 7, 9, 12, 18]. $I^{2}$ was 0.00, indicating that the heterogeneity of these studies was small. Funnel plots and regression-based Egger test showed no evidence of publication bias (*p* = 0.97) (Fig. 3B). Meta analysis with a fixed model revealed that there was no significant difference between the JWT and JBT (*p* = 0.20). The aggregated OR value was 2.33 (Fig. 5).

#### 3.3.3 MI

**Fig. 6.**
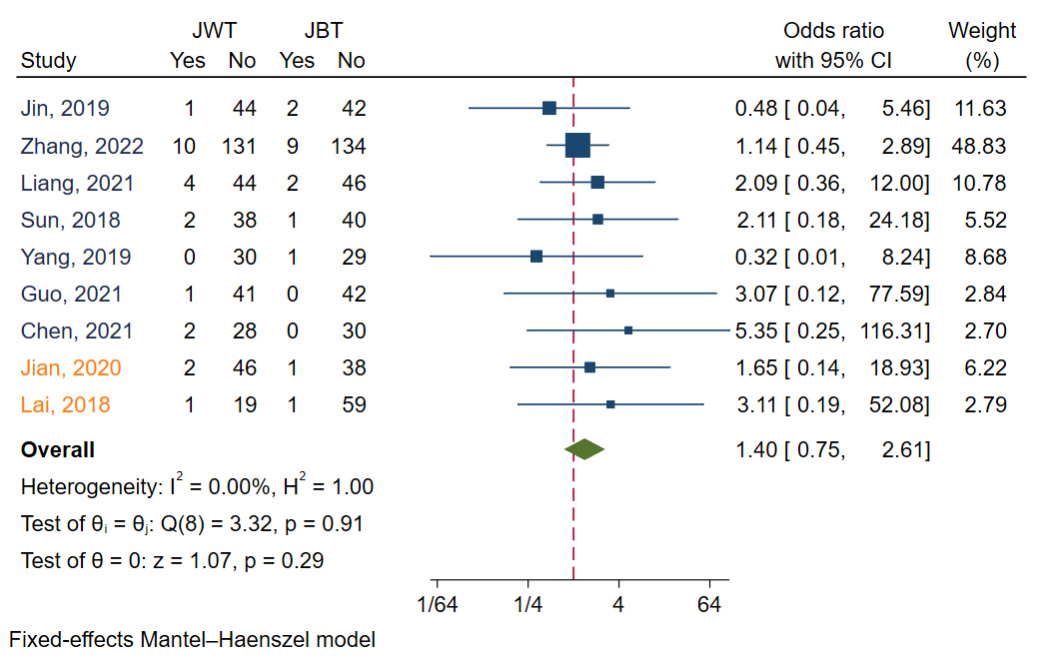
**Forest plot for comparisons of MI between JBT and JWT**. MI, Myocardial infarction; JBT, jailed balloon technique; JWT, jailed wire technique. Studies in blue font are randomized controlled trails, while those in orange font are observational studies.

MI was reported in nine studies [6, 7, 9, 11, 12, 13, 14, 15, 18]. $I^{2}$ was 0.00, indicating that the heterogeneity of these studies was small. Funnel plots and regression-based Egger test showed no evidence of publication bias (*p* = 0.62) (Fig. 3C). Meta analysis with a fixed model revealed that there was no significant difference between the JWT and JBT (*p* = 0.29). The aggregated OR value was 1.40 (Fig. 6).

#### 3.3.4 TLR

**Fig. 7.**
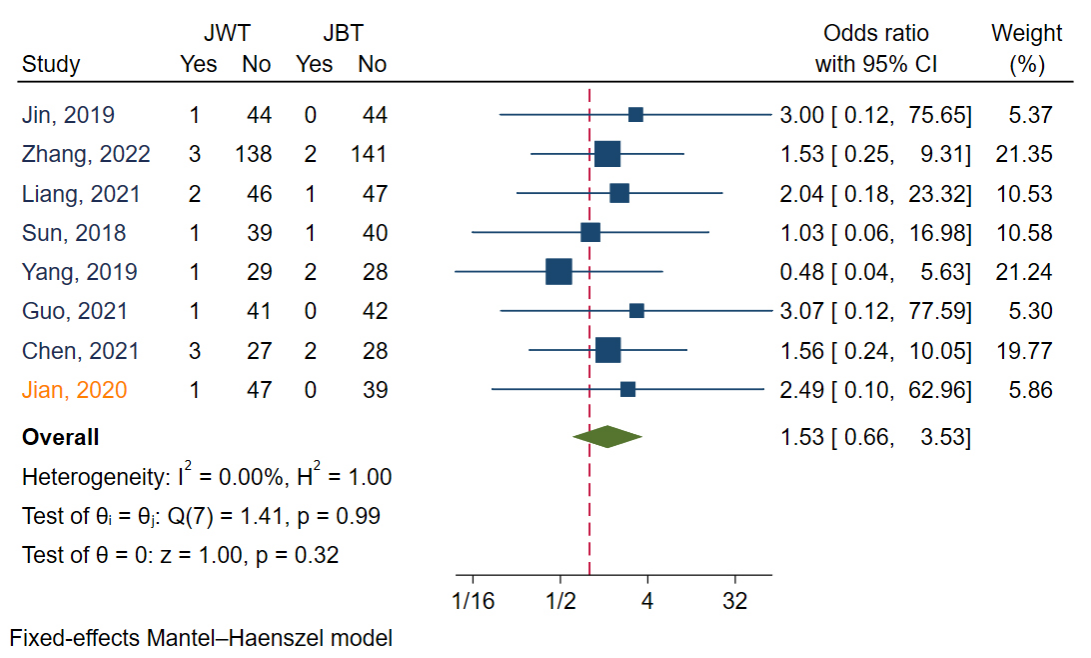
**Forest plot for comparisons of TLR between JBT and JWT**. TLR, target lesion revascularization; JBT, jailed balloon technique; JWT, jailed wire technique. Studies in blue font are randomized controlled trails, while that in orange font is an observational study.

TLR was reported in eight studies [6, 7, 9, 11, 12, 13, 14, 15]. $I^{2}$ was 0.00, indicating that the heterogeneity of these studies was small. Funnel plots and regression-based Egger test showed no evidence of publication bias (*p* = 0.72) (Fig. 3D). Meta analysis with a fixed model revealed that there was no significant difference between the JWT and JBT (*p* = 0.32). The aggregated OR value was 1.53 (Fig. 7).

### 3.4 Secondary Endpoints

#### 3.4.1 Side Branch Occlusion

**Fig. 8.**
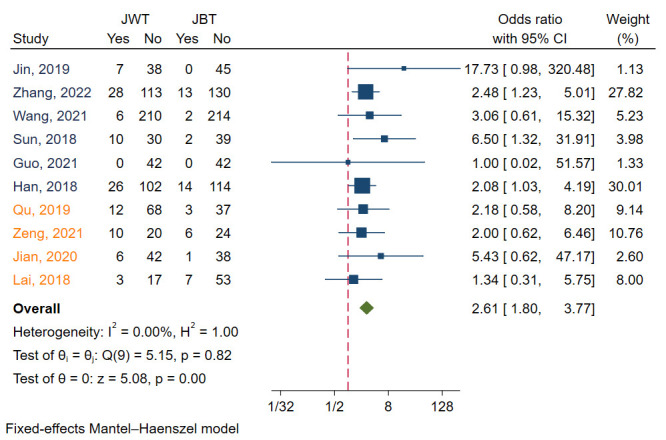
**Forest plot for comparisons of side branch occlusion between JBT and JWT**. JBT, jailed balloon technique; JWT, jailed wire technique. Studies with label of blue font were randomized controlled trails, while orange font were observational studies.

SB occlusion was reported in ten studies [6, 7, 8, 10, 11, 12, 14, 16, 17, 18]. $I^{2}$ was 0.00, indicating that the heterogeneity of these studies was small. Funnel plots and regression-based Egger test showed no evidence of publication bias (*p* = 0.36) (Fig. 3E). Meta analysis with a fixed model revealed that JBT had a significantly lower SB occlusion compared with JWT (*p* = 0.00). The aggregated OR value was 2.61 (Fig. 8).

#### 3.4.2 SB Dissection

**Fig. 9.**
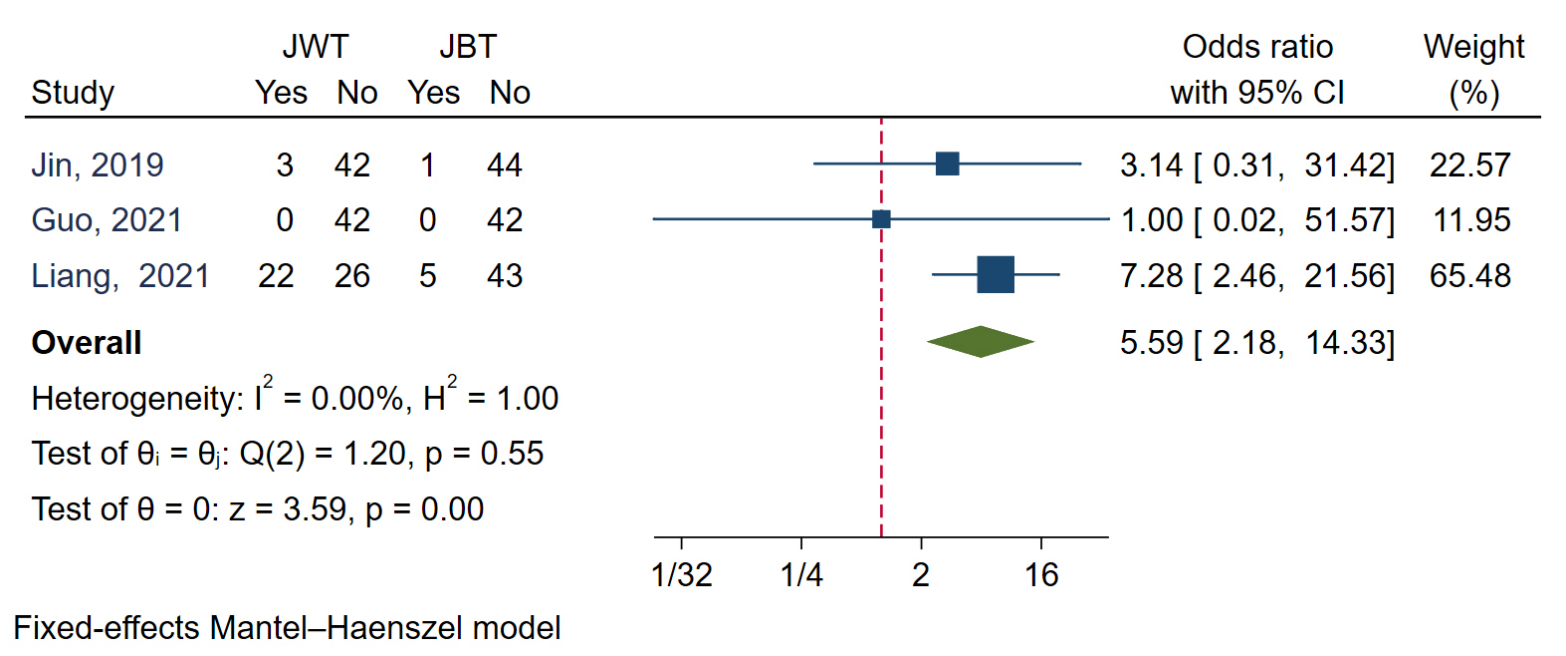
**Forest plot for comparisons of side branch dissection between JBT and JWT**. JBT, jailed balloon technique; JWT, jailed wire technique. Studies with label of blue font were randomized controlled trails.

SB dissection was reported in three studies [6, 9, 14]. $I^{2}$ was 0.00, indicating that the heterogeneity of these studies was small. Funnel plots and regression-based Egger test showed no evidence of publication bias (*p* = 0.27) (Fig. 3F). Meta analysis with a fixed model revealed JBT had a significantly lower SB dissection rate compared with JWT (*p* = 0.00). The aggregated OR value was 5.59 (Fig. 9).

## 4. Discussion

Coronary bifurcation lesions constitute 15–20% of percutaneous coronary interventions. SB occlusion caused by carina or plaque shift could lead to perioperative MI or death. JWT has been recommended by the EBC as a routine maneuver to prevent SB occlusion or facilitate rescuing a compromised SB. However, clinical practice revealed that the preventative effect of JWT was limited. JBT appears to decrease SB occlusion, but it has not been widely accepted in clinical practice due to a lack of clinical studies.

As far as we know, this is the first systematic review and meta-analysis to compare the JWT and JBT. In this present review, 13 studies were reviewed and six endpoints were analyzed. The heterogeneity between these studies was small. No evidence of publication bias was observed. According to the results, the individual rates of cardiac death, MI, and TLR were not significantly different between the techniques. However, the composite of MACE, as well as the secondary endpoints of SB occlusion and dissection were significantly lower in the JBT group. However, JWT is favored for its lower rates of dissection in clinical practice. In the literature, JBT has been shown to increase the risk of dissection, especially when the jailed balloon is inflated [19, 20]. Since these reports were single-arm studies, they were not enrolled in this review. We can conclude from this meta-analysis that JBT is a more effective and safer technique by reducing both procedural SB occlusion and long-term MACE.

The reasons that JBT performed better than JWT include: (1) larger spatial occupation to prevent carina or plaque shift; (2) prompt dilation to restore SB flow once it was compromised; and (3) angle modifier facilitating SB rewiring.

Another issue we should be concerned with is balloon entrapment. Through searching the relative literature, we only find one case of balloon entrapment in a calcified lesion [21], but lots of wire entrapment when adopting JWT. Balloon entrapment never happened in our own experience. This might attribute to hydrophilic coating of a balloon. Furthermore, we could retrieve the jailed balloon after successive maneuvers of inflating and deflating it.

After Burzotta *et al*. [3] proposed JBT in 2010, it was further modified by different versions, such as modified JBT, jailed semi-inflation technique, balloon stent kissing technique and so on [22, 23, 24]. They differed mainly in the balloon positions, inflation pressure and timing. It was still not clear which version performed best. However, results of this meta-analyis would suggest that JBT was better than JWT. Further clinical evidences and net-work meta-analysis are needed to compare the different JBTs.

## 5. Limitations

The main limitations of this study are: (1) the sample size was relatively small; (2) the definitions of endpoints varied across different studies; and (3) the techniques of JBT differed amongst the studies.

## 6. Conclusions

JBT can prevent SB occlusion more effectively and did not cause any increase in immediate or long-term complications. JBT or its modified versions might be the technique of choice in treating those SBs with a high risk of occlusion.

## Data Availability

All Template data collection forms, data extracted from included studies, data used for all analyses, and analytic code in the review are publicly available.

## Abbreviations

SB, side branch; MV, main vessel; JWT, jailed wire technique; EBC, European bifurcation club; JBT, jailed balloon technique; RCT, randomized controlled trials; MACE, major adverse cardiac events; MI, myocardial infarction; TLR, target lesion revascularization; TIMI, thrombolysis in myocardial infarction; OCT, optical coherence tomography; IVUS, intravascular ultrasound; NOS, Newcastle-Ottawa Quality Assessment Scale.

## Supplementary Material

Supplementary material associated with this article can be found, in the online version, at https://doi.org/10.31083/j.rcm2503107.

## References

[1] Lassen JF, Burzotta F, Banning AP, Lefèvre T, Darremont O, Hildick-Smith D (2018). Percutaneous coronary intervention for the left main stem and other bifurcation lesions: 12th consensus document from the European Bifurcation Club. *EuroIntervention*.

[2] Hahn JY, Chun WJ, Kim JH, Song YB, Oh JH, Koo BK (2013). Predictors and outcomes of side branch occlusion after main vessel stenting in coronary bifurcation lesions: results from the COBIS II Registry (COronary BIfurcation Stenting). *Journal of the American College of Cardiology*.

[3] Burzotta F, Trani C, Sianos G (2010). Jailed balloon protection: a new technique to avoid acute side-branch occlusion during provisional stenting of bifurcated lesions. Bench test report and first clinical experience. *EuroIntervention*.

[4] Sterne JAC, Savović J, Page MJ, Elbers RG, Blencowe NS, Boutron I (2019). RoB 2: a revised tool for assessing risk of bias in randomised trials. *British Medical Journal*.

[5] Stang A (2010). Critical evaluation of the Newcastle-Ottawa scale for the assessment of the quality of nonrandomized studies in meta-analyses. *European Journal of Epidemiology*.

[6] Jin Z, Song L, Zheng Z, Zhang S, Wang M (2019). Balloon-stent kissing technique versus jailed wire technique for interventional treatment of coronary bifurcation lesions: Comparison of short- and long-term clinical outcomes. *Medicine*.

[7] Zhang D, Zhao Z, Gao G, Xu H, Wang H, Liu S (2022). Jailed Balloon Technique Is Superior to Jailed Wire Technique in Reducing the Rate of Side Branch Occlusion: Subgroup Analysis of the Conventional Versus Intentional StraTegy in Patients With High Risk PrEdiction of Side Branch OccLusion in Coronary Bifurcation InterVEntion Trial. *Frontiers in Cardiovascular Medicine*.

[8] Wang M, Liu H, Xu B, Bian X, Liu L, Hu F (2021). A Randomized Controlled Study on the Efficacy of Protective Ballooning Technique for the Prevention of Side Branch Occlusion in Coronary Non-left Main Bifurcation Lesions. *Chinese Circulation Journal*.

[9] Liang G, Luo J, Liao G, Yang C, Zhai Y (2021). Clinical Research of Jailed Ballon Protection Technique in Interventional Treatment of Non-left Main Coronary Artery Bifurcation. *Chinese Journal of Integrative Medicine on Cardio-Cerebrovascular Disease*.

[10] Zeng Q, Xia J, Hu X, Li K, Xiong Y (2021). Clinical application of detention balloon technique (JBT) in interventional treatment of bifurcation of coronary artery. *Contemporary Medicine*.

[11] Sun X, Li X, Yang L, Zhang H, Li Y (2018). Comparison of effect of jailed balloon technique and double guide wire technique on protection of branch opening in treatment of bifurcation lesion in coronary artery. *Anhui Medical Journal*.

[12] Jian X, Fan Z, Ji H, Li L, Liu T, Cardiology DO (2020). Safety and influence on short-term and mid-term prognosis of jailed balloon technique in treatment of bifurcation lesions with emergency percutaneous coronary intervention in patients with acute myocardial infarction. *Chinese Journal of Evidence-Based Cardiovascular Medicine*.

[13] Yang Z, Li X, Yan T, Fu W (2019). Application of detention balloon technique in bifurcation lesions of non-left main trunk. *Journal of Frontiers of Medicine*.

[14] Guo S, Zhang O, Zeng X (2021). Application of detention balloon technique in bifurcation lesions of non-left main trunk. *Chinese Journal of Practical Medicine*.

[15] Chen W, Liang J, Liang L (2021). Analysis on the role of jailed balloon technique in the vascular protection of percutaneous coronary intervention for coronary bifurcation lesions in the elderly. *China Medicine and Pharmacy*.

[16] Han H, Huang H, Luo P, Li J, Tian W, Chen Y (2018). Short-term Outcome of Jailed Balloon Technique and Jailed Wire Technique in the Intervention of True Coronary Bifurcation Lesions. *Sichuan Medical Journal*.

[17] Qu WB, Zhang W, Liu JY, Zhang F, Mu SN, Zhang SM (2019). Modified balloon-stent kissing technique avoid side-branch compromise for simple true bifurcation lesions. *BMC Cardiovascular Disorders*.

[18] Lai JX, Mo ZQ, Song AJ, Tan WF (2018). Clinical efficacy of different side branch protection techniques on patients receiving coronary intervention and prognostic analysis. *European Review for Medical and Pharmacological Sciences*.

[19] Nomura T, Wada N, Ota I, Tasaka S, Ono K, Sakaue Y (2021). Inflation Pressure in Side Branch during Modified Jailed Balloon Technique Does Not Affect Side Branch Outcomes. *Journal of Interventional Cardiology*.

[20] Ermiş E, Uçar H, Demirelli S, İpek E, Gür M, Çaylı M (2018). Assessment of side branch patency using a jailed semi-inflated balloon technique with coronary bifurcation lesions. *Turk Kardiyoloji Dernegi Arsivi: Turk Kardiyoloji Derneginin Yayin Organidir*.

[21] Numasawa Y, Hitomi Y, Imaeda S, Yokokura S, Tanaka M, Tabei R (2019). Stent Deformation Caused by Entrapment of the Side Branch Balloon Catheter During the Jailed Balloon Protection Technique for a Calcified Coronary Bifurcation Lesion: A Case Report and Literature Review. *Cardiovascular Revascularization Medicine: Including Molecular Interventions*.

[22] Saito S, Shishido K, Moriyama N, Ochiai T, Mizuno S, Yamanaka F (2018). Modified jailed balloon technique for bifurcation lesions. *Catheterization and Cardiovascular Interventions*.

[23] Çaylı M, Şeker T, Gür M, Elbasan Z, Şahin DY, Elbey MA (2015). A Novel-Modified Provisional Bifurcation Stenting Technique: Jailed Semi-Inflated Balloon Technique. *Journal of Interventional Cardiology*.

[24] Jin Z, Li L, Wang M, Zhang S, Chen Z, Shen Z (2013). Innovative provisional stenting approach to treat coronary bifurcation lesions: balloon-stent kissing technique. *The Journal of Invasive Cardiology*.

